# Omega-3 Fatty Acid Supplementation Improves Heart Rate Variability in Obese Children

**DOI:** 10.1155/2018/8789604

**Published:** 2018-02-26

**Authors:** Christoph Baumann, Ulla Rakowski, Reiner Buchhorn

**Affiliations:** ^1^Medical Faculty, University of Wuerzburg, Josef-Schneider-Straße 2, Würzburg, Germany; ^2^Caritas-Krankenhaus Bad Mergentheim, Department of Pediatrics, Uhlandstraße 7, Bad Mergentheim, Germany

## Abstract

Obese children and adolescents are at high risk of developing cardiovascular diseases later in life. We hypothesized that cardiovascular prophylaxis with omega-3 fatty acids could benefit them. In our study, 20 children and adolescents (mean body mass index percentile: 99.1; mean age: 11.0 years) underwent two ambulatory 24 h Holter electrocardiography (ECG) recordings (before and after at least 3 months of omega-3 fatty acid supplementation). Time domain heart rate variability (HRV) and heart rate (HR) were examined for these patients. As a control, we used 24 h Holter ECG recordings of 94 nonobese children and adolescents. Time domain HRV parameters, which are indicators of vagal stimulation, were significantly lower in obese patients than in healthy controls, but HR was higher (standard deviation of the normal-to-normal [SDNN] interbeat intervals: −34.02%; root mean square of successive differences [RMSSD] between normal heartbeats: −40.66%; percentage of consecutive RR intervals [pNN50]: −60.24%; HR: +13.37%). After omega-3 fatty acid supplementation, time domain HRV parameters and HR of obese patients were similar to the values of healthy controls (SDNN interbeat intervals: −21.73%; RMSSD: −19.56%; pNN50: −25.59%; HR: +3.94%). Therefore, omega-3 fatty acid supplementation may be used for cardiovascular prophylaxis in obese children and adolescents.

## 1. Introduction

Obesity is one of the most prevalent and serious health problems for children and adolescents. During childhood and adolescence, obesity leads to severe sequelae such as increased blood pressure, glucose intolerance, fat metabolism disorders, back pain, hepatic steatosis, dermatoses, and mental illnesses. These are much more common in obese children and adolescents than in their healthy peers. Furthermore, these children are at high risk of remaining overweight in adulthood and of developing chronic cardiovascular diseases, such as coronary heart disease, later in life. Despite the obvious relationship between increased body mass index (BMI) and the development of cardiovascular diseases, the reasons for this association remain vague. It has been suggested that the development of cardiovascular diseases involves various factors such as hypertension, insulin resistance, altered lipid profile, and impairment of cardiac autonomic control [1].

In recent studies, omega-3 fatty acids have had a positive effect on these factors. Evidence suggests that omega-3 fatty acids reduce blood triglyceride levels, lower blood pressure, increase insulin sensitivity, and improve heart rate variability (HRV) parameters, which are possible markers of cardiovascular health [2–5].

Therefore, omega-3 fatty acid supplementation may be a low-cost intervention with few side effects for obese children and adolescents that could decelerate the development of chronic cardiovascular diseases. In this study, we investigated the effects of omega-3 fatty acid supplementation on HRV and heart rate (HR) of obese children and adolescents for whom conventional treatment approaches have failed.

## 2. Patients and Methods

In the context of a superordinate project that examines standard HRV values in hospitalized patients, we analyzed HRV in obese patients to gain a better understanding of the autonomic nervous system. The project to survey standard HRV values was approved by the Landesärztekammer Baden-Württemberg (F-2012-056).

The study did not carry a risk of harm to human subjects. Recorded Holter electrocardiography (ECG) results during this study were part of the standard examination procedure for obese children and adolescents at our outpatient clinic. Omega-3 fatty acid as a dietary supplement is considered safe in the administered doses. We further conducted our study in compliance with the Declaration of Helsinki.

### 2.1. Patients

The current study included 20 European children and adolescents with a BMI above the 95th percentile and an average age of 11.0 years (patient characteristics are summarized in Table 1). All patients were referred to the pediatric outpatient clinic of the Caritas Hospital Bad Mergentheim between October 2013 and May 2017 for further diagnostics and therapy for obesity. All study participants and their parents or guardians provided informed consent. No patients received relevant medication during ECG recordings.

### 2.2. Control Group

As controls, values of healthy nonobese children (*N* = 94) from a previous project were used. The HRV values of our controls were in accordance with the calculated normal values from a Polish study [6].

### 2.3. Design

Obese children and adolescents were screened for metabolic syndrome according to the criteria of the Identification and Prevention of Dietary- and Lifestyle-Induced Health Effects in Children and Infants (IDEFICS) and for autonomic dysfunction by 24 h Holter ECG analysis at our outpatient clinic. In addition to implementing diet and lifestyle changes, we recommended that our patients should use omega-3 fatty acid supplements. All patients included in the study followed our advice and used fish oil capsules that contained at least 400 mg eicosapentaenoic acid (EPA) and 120 mg docosahexaenoic acid (DHA) daily. At a follow-up appointment after an average of 214 days, results of a second 24 h Holter ECG were recorded and analyzed. Additionally, height and weight were measured for each patient, and the BMI and BMI percentiles were calculated.

### 2.4. 24-Hour ECG and Analysis of Heart Rate Variability

During our study, time domain HRV parameters and HR were dependent variables. HRs and HRV parameters have been shown to be predictors of sudden cardiac death and mortality after myocardial infarction. Moreover, several obesity-related conditions, such as type 2 diabetes, sleep apnea, depression, hypertension, coronary heart disease, congestive heart failure, and cerebrovascular events, seem to be linked to decreased HRV and increased HR. In contrast to HR, HRV parameters have been shown to be more sensitive to changes in the autonomic nervous system and to precede HR trends [7].

To record ECG results, we used a two-channel Holter monitor (Pathfinder; Spacelabs Healthcare GmbH, Nürnberg, Germany). Children and adolescents were not placed on bed rest; instead, they followed their usual daily routines. The Holter ECG recordings were analyzed as average values of all data collected during 24 h. Measurement and interpretation of HRV parameters in the sample were standardized according to the currently established guidelines. The following time domain HRV variables were analyzed: the standard deviation of the normal-to-normal (SDNN) interbeat intervals, root mean square of successive differences (RMSSD) between normal heartbeats, and percentage of consecutive RR intervals (pNN50).

In contrast to frequency domain parameters, time domain HRV parameters are easily calculated and do not require complex computations. Moreover, they require less stationarity than most frequency domain parameters and are suitable for long-term HRV analyses.

### 2.5. Statistical Analysis

We used SPSS version 23.0 for all statistical analyses. All results were reported as mean ± standard deviation (SD). Parametric statistics were used for all comparisons because most variables were normally distributed. Patients were compared to controls using an independent samples *t*-test for equality of means, and they were compared before and after omega-3 fatty acid supplementation using a paired samples *t*-test for equality of means. A *p* value < 0.05 was considered statistically significant. We performed a post hoc power analysis for the SDNN parameter using *G*^*∗*^Power 3.1. With *α* set at .05 and a sample size of 20, we calculated the statistical power (1 − *β*) as 0.98.

## 3. Results

Patient characteristics and 24 h mean HRV values are summarized in Table 1. At the first appointment, the BMI of the 20 obese children and adolescents was 68.36% higher than that of healthy controls. In addition, HR was high and HRV parameters were low compared to those of healthy controls. Between the first examination appointment and the follow-up appointment, the mean BMI decreased by 0.67%. However, this change was not statistically significant, and all patients maintained a BMI higher than the 95th percentile. In contrast to the statistically insignificant decrease in mean BMI, all HRV parameters increased significantly after omega-3 fatty acid supplementation. SDNN interbeat intervals increased by 19.34%, RMSSD increased by 34.93%, and pNN50 increased by 81.37%.

Additionally, the HR of the 20 obese children and adolescents were approximately the same as those of healthy controls. Mean HR decreased from 95.0 to 87.2 bpm (*p* < 0.0001). The mean HR decreased by 8-9 bpm and was comparable during the day and night.

## 4. Discussion

HRV is a physiological phenomenon. The instantaneous HR and its variation are the results of several different inputs at the sinus node area. The main inputs arise from the sympathetic nervous system and the parasympathetic system. However, both systems are influenced by subordinate factors such as hormones, sleep-wake cycle, stress, physical activity, respiration, or thermoregulation. The primary process of the sympathetic nervous system is to activate the body's fight-or-flight response, whereas the parasympathetic system is responsible for the activation of resting and digesting activities. From a biochemical point of view, HRV analysis is based on the differences in neurotransmission in the parasympathetic and sympathetic systems. While altered parasympathetic activity directly increases or decreases the HR, the sympathetic nervous system reacts relatively slowly. The sympathetic varicosities and fibers mainly secrete norepinephrine, which has to reach its receptors by diffusion and then acts through a second messenger pathway. Unlike parasympathetic axonal endings, the sympathetic varicosities are not assigned to postsynaptic terminals. Moreover, the adrenal gland produces norepinephrine and epinephrine and releases them into the bloodstream, as part of the sympathetic nervous system. The signals of the sympathetic nervous system are then terminated, predominantly by the reuptake of neurotransmitters or enzymatic cleavage. In contrast, the postganglionic fibers of the parasympathetic nervous system regulate the HR rapidly via the release of acetylcholine and the subsequent signal cascade. Acetylcholine is cleaved and the signal is terminated via esterase [8].

For all time domain parameters, high values presumably represent a strong parasympathetic tone or little sympathetic activity. In this study, we showed that supplementation of omega-3 fatty acids increased the time domain parameters and decreased HR in obese children and adolescents. Accordingly, the most common parameter in the HRV literature, SDNN interbeat intervals, normalized and only slightly differed from the values of healthy controls after omega-3 fatty acid supplementation (108.6 ms versus 129.6 ms). In a similar manner, the other two time domain parameters, RMSSD and pNN50, increased significantly after omega-3 fatty acid supplementation. Our findings consequently indicated that omega-3 fatty acid supplementation may have a positive effect on cardiovascular health in obese children. The observed changes in HRV parameters in this study may be favorable because low HRV parameters and high HR have been shown to be related to cardiovascular diseases and several other comorbidities associated with obesity [9–13]. On the contrary, a high level of HRV and moderate HR within an organism reflect cardiac health, self-regulatory capacity, and adaptability [7].

The observed effects of omega-3 fatty acids on heart rate and HRV and HR may be due to the three mechanisms:

1. It is possible that omega-3 fatty acids directly influence the voltage-gated ion channels in the sinus node area [14]. This theory is supported by a study that investigated the HR of cardiac transplant recipients before and after omega-3 fatty acid supplementation. In their study, Harris et al. showed that omega-3 fatty acid supplementation reduces HR in cardiac transplant recipients who lack cardiac vagal innervation [15]. These findings imply that omega-3 fatty acids may modify the electrophysiology of the cardiac conduction system. Through this mechanism, omega-3 fatty acids could also lower the intrinsic HR of obese children and adolescents. Due to the nonlinear relationship between HRV and RR interval, HRV estimated from RR intervals is negatively correlated with HR. Consequently, decreased HR would also be accompanied by increased HRV [16].
2. Improvements in HRV and HR may be linked to the effects of omega-3 fatty acids on insulin sensitivity. Omega-3 fatty acids have been shown to change insulin signaling pathways and gene expression [17]. In a recent animal study, supplementation of omega-3 fatty acids reversed the effects of a high-carbohydrate diet on insulin sensitivity [18]. Although it has been suggested that diabetic autonomic neuropathy damages the vagus nerve, which physiologically controls the heart rate and the secretion of insulin from beta cells, autonomic dysfunction in the wake of insulin resistance is also possible. Bergholm et al. showed that insulin possibly modulates autonomic tone [19]. Eventually, omega-3 fatty acids, which have been shown to increase insulin sensitivity in patients with metabolic disorders, may shift this relationship and alter autonomic regulation, thereby changing HRV parameters, which measure the activity of the autonomic nervous system [20].
3. Moreover, it is possible that omega-3 fatty acid supplementation changed HRV parameters and HR due to their effects on vascular function and inflammation. Dangardt et al. showed that omega-3 supplementation decreased lymphocytes, monocytes, and the level of proinflammatory cytokines in obese adolescents. The decrease in these proinflammatory factors may be linked to withdrawal of the sympathetic overdrive [21]. HRV, which is inversely related to levels of inflammatory markers, may capture these changes in the immune system [22]. Additionally, omega-3 fatty acids lower the systemic vascular resistance through changes in endothelial function, which can result in lower blood pressure and higher HRV levels in patients with risk factors for cardiovascular diseases [3].

## 5. Limitations

The present study had several methodological limitations. First, we cannot state with certainty whether omega-3 fatty acids were responsible for the observed HR and HRV changes. Given that the intervals between the two Holter ECG measurements were relatively long, aging may have decreased HR and increased HRV. However, a clinical study showed that aging cannot explain a decrease of 8.32% [23]. Only a decrease of 2%-3% in HR within 1 year would be physiologically meaningful. Second, a more important limitation of our study was that some of our patients lost weight. Although the decrease in BMI was minimal, weight loss could have caused the observed changes in HR and HRV. It has been observed that weight loss leads to sympathetic withdrawal and parasympathetic activation. Third, the sample used in the present study was relatively small (*N* = 20). Due to the study design, we could not control or detect possible confounders such as movement or lifestyle changes. Fourth, the study involved a single center. Hence, generalization of the results was difficult. Fifth, the use of HRV for risk stratification of children and adolescents has not been investigated. However, we showed that HRV may be an interesting biological marker for assessing the effects of omega-3 fatty acid supplementation in obese children and adolescents.

## 6. Conclusion

Despite the methodological limitations and shortcomings, this study offered preliminary data illustrating that omega-3 fatty acids may favorably influence the cardiovascular health of obese children and adolescents. Future studies should implement randomized, placebo-controlled designs and adhere to a strict protocol regarding the measurement point of HRV because we cannot rule out confounders that potentially influence HRV. In conclusion, because severe side effects do not occur with the use omega-3 fatty acid supplements at the recommended dose, we recommend them as possible cardiovascular prophylaxis for obese children and adolescents.

## Figures and Tables

**Table 1:** Demographic data and 24 h heart rate analysis data of obese children before and after omega-3 fatty acid supplementation.

Parameter	Healthy controls	Baseline		Omega-3 fatty acids		Baseline versus omega-3 fatty acids *p* value^2^
	Mean ± SD	Mean ± SD	*p* value^1^	Mean ± SD	*p* value^1^	
	*N* = 94	*N* = 20		*N* = 20		
Age [years]	11.3 ± 2.7	11.0 ± 4.6	*ns*	11.6 ± 3.8	*ns*	**ns**
Height [cm]	148.2 ± 17.6	153.4 ± 22.0	*ns*	155.9 ± 20.5	*ns*	**0.0035**
BMI [kg/m^2^]	17.7 ± 2.8	29.8 ± 4.6	*<0.0001*	29.6 ± 4.1	*<0.0001*	**ns**
BMI percentile [%]	44.5 ± 26.2	99.1 ± 1.5	*<0.0001*	99.1 ± 1.3	*<0.0001*	**ns**
24 h heart rate [bpm]	83.8 ± 12.5	95.0 ± 8.9	*<0.0001*	87.2 ± 11.3	*ns*	**<0.0001**
HR, day [bpm]	93.3 ± 13.5	104.4 ± 9.3	*0.0009*	95.7 ± 11.2	*ns*	**0.0002**
HR, night [bpm]	70.7 ± 9.2	83.9 ± 9.3	*<0.0001*	75.8 ± 12.1	*0.035*	**0.0017**
RMSSD [ms]	45.5 ± 12.1	27.2 ± 9.4	*<0.0001*	36.7 ± 16.2	*0.0063*	**0.0008**
pNN50 [%]	25.4 ± 10.8	10.2 ± 8.3	*<0.0001*	18.5 ± 13.6	*0.014*	**0.0009**
SDNN [ms]	166.1 ± 44.6	108.6 ± 25.1	*<0.0001*	129.6 ± 32.4	*0.0007*	**0.0043**

^1^Unpaired *t*-test used for healthy controls and baseline/omega-3 fatty acids (italic). ^2^Paired *t*-test used for baseline and omega-3 fatty acids (bold). ns = not significant.

## Data Availability

The datasets analyzed in this study are available from Reiner Buchhorn or Christoph Baumann on reasonable request.
